# Re-assessing gallium-67 as a therapeutic radionuclide^☆^

**DOI:** 10.1016/j.nucmedbio.2016.10.008

**Published:** 2017-03

**Authors:** Muhamad F. bin Othman, Nabil R. Mitry, Valerie J. Lewington, Philip J. Blower, Samantha Y.A. Terry

**Affiliations:** a King's College London, Department of Imaging Chemistry and Biology, St. Thomas' Hospital, London, SE1 7EH, UK; b Guy's & St Thomas' NHS Foundation Trust, Nuclear Medicine Department, London, SE1 9RT, UK

**Keywords:** Gallium-67, Radionuclide therapy, Auger electrons, Clonogenic assay

## Abstract

**Introduction:**

Despite its desirable half-life and low energy Auger electrons that travel further than for other radionuclides, ^67^Ga has been neglected as a therapeutic radionuclide. Here, ^67^Ga is compared with Auger electron emitter ^111^In as a potential therapeutic radionuclide.

**Methods:**

Plasmid pBR322 studies allowed direct comparison between ^67^Ga and ^111^In (1 MBq) in causing DNA damage, including the effect of chelators (EDTA and DTPA) and the effects of a free radical scavenger (DMSO). The cytotoxicity of internalized (by means of delivery in the form of oxine complexes) and non-internalized ^67^Ga and ^111^In was measured in DU145 prostate cancer cells after a one-hour incubation using cell viability (trypan blue) and clonogenic studies. MDA-MB-231 and HCC1954 cells were also used.

**Results:**

Plasmid DNA damage was caused by ^67^Ga and was comparable to that caused by ^111^In; it was reduced in the presence of EDTA, DTPA and DMSO. The A_50_ values (internalized activity of oxine complexes per cell required to kill 50% of cells) as determined by trypan blue staining was 1.0 Bq/cell for both ^67^Ga and ^111^In; the A_50_ values determined by clonogenic assay were 0.7 Bq/cell and 0.3 Bq/cell for ^111^In and ^67^Ga respectively. At the concentrations required to achieve these uptake levels, non-internalized ^67^Ga and ^111^In caused no cellular toxicity. Qualitatively similar results were found for MDA-MB-231 and HCC1954 cells.

**Conclusion:**

^67^Ga causes as much damage as ^111^In to plasmid DNA in solution and shows similar toxicity as ^111^In at equivalent internalized activity per cell. ^67^Ga therefore deserves further evaluation for radionuclide therapy.

**Advances in knowledge and implications for patient care:**

The data presented here is at the basic level of science. If future in vivo and clinical studies are successful, ^67^Ga could become a useful radionuclide with little healthy tissue toxicity in the arsenal of weapons for treating cancer.

## 1 Introduction

Radiopharmaceutical therapies, such as ^131^I–MIBG, anti-CD20 antibodies (labeled with ^90^Y or ^131^I), and ^177^Lu-Octreotate, have become standard in the clinic. These beta particle-emitting treatments, however, are generally not curative and can cause toxicity to healthy tissue due to the long range (up to 1 cm for ^90^Y) by high beta energies. Radioisotopes emitting Auger electrons with a much shorter range (<1 μm) are now being considered for targeted radionuclide therapy and could become useful tools in targeting micrometastases that play a detrimental role in tumor recurrence.

Gamma camera imaging with ^67^Ga has been used regularly in the clinic since the 1980s to image lymphoma where it was useful in disease staging, monitoring disease progression and relapse, and predicting therapy response [1]. In Hodgkin's disease, the detection sensitivity is 70 to 83% [2]. In non-Hodgkin's lymphoma the detection sensitivity depends on cell differentiation status; less differentiated cells show higher avidity for gallium [1]. Other gallium-avid cancers include lung cancer, melanoma and multiple myeloma [1], [2]. In these applications ^67^Ga is administered as ^67^Ga-citrate and ^67^Ga uptake by cells is believed to be transferrin-mediated [3], although there is also evidence for transferrin-independent mechanisms [4], [5], [6].

A feature of ^67^Ga is that besides its gamma emissions for scintigraphy and SPECT imaging, it also emits Auger electrons [7] and thus has potential as a therapeutic radionuclide. As such it could form part of a “theranostic pair” with the generator-produced positron emitter ^68^Ga. Despite producing fewer Auger electrons per decay (average of 4.7) than fellow Auger electron emitter ^111^In (14.7), the average total Auger electron energy released per decay of ^67^Ga (6.3 keV) is similar to that of ^111^In (6.8 keV) [8]. In fact, amongst Auger electron emitters, ^67^Ga produces amongst the most energetic (7 to 9 KeV) and longest ranging (up to 2.4 μm in water) Auger electrons [7]. This may reduce the need for the radionuclide to be localized in specific subcellular compartments in order to be effective.

^67^Ga has been explored previously, to a limited extent, as a radionuclide for therapeutic applications [9], [10], [11], [12], [13]. In vitro results were promising and showed that treatment with ^67^Ga diminished clonogenic capacity in human U937 lymphoma cells [9] and in myeloid leukemic blasts from acute myeloid leukemia patients [10]. A feasibility study in eight patients with relapsed acute leukemia was less successful due to the low cell uptake of ^67^Ga-citrate [11], which might have arisen in part from using higher citrate concentrations in the clinical preparations than used in the in vitro work. Others have explored the therapeutic potential of ^67^Ga in lymphoma when coupled to anti-CD74 antibodies [12], [14] or anti-LL1 antibodies [13]. Michel et al. showed that ^67^Ga was two to three times more potent than ^111^In when coupled to anti-CD74 antibodies on the basis of equivalent total disintegrations in the medium per viable cell [12]. Low specific activities and lack of purpose-designed gallium chelators and conjugates at that time, however, led to a lack of further enquiry in this field.

In recent years the development of peptides and proteins labeled with ^68^Ga, arising from the growing popularity of the ^68^Ga generator for positron emission tomography applications, has led to a new generation of effective bifunctional chelators for gallium [15], [16], [17], [18], [19], [20], [21], [22], [23], [24], [25]. For example, the trishydroxypyridinone chelator allows radiolabelling of molecular targeting agents with gallium using fast and simple one-step procedures [15], [20]. The resulting enhanced versatility and range of potentially useful targeting agents now presents an opportunity to reconsider ^67^Ga as a targeted therapeutic radionuclide.

Here a comparison is made between ^67^Ga and the well-characterized and clinically evaluated radionuclide ^111^In, which has been successfully tested preclinically as a therapeutic radionuclide attached to cell surface or intracellular targets [26], [27], [28]. ^67^Ga and ^111^In have similar half-lives (78 and 67 h, respectively) and both produce gamma rays. We used the cell-free pBR322 plasmid assay to directly compare DNA damage induced by the radionuclides without complications due to cellular and subcellular barriers, DNA repair mechanisms and other cellular responses. For the first time, the levels of activity per cell required to achieve significant cytotoxic effects was calculated from viability and clonogenic assays using radiolabelled lipophilic complexes in three prostate and breast cancer cell lines, selected for their possible future use in in vivo models for radionuclide therapy of circulating tumor cells and micrometastases using Auger electron emitters.

## 2 Materials and methods

### 2.1 Radionuclide preparation

^111^In-chloride (^111^InCl_3_) (Mallinckrodt, Netherlands) was supplied at 111 MBq in 0.3 mL 0.05 M HCl. ^67^Ga-citrate (6.46 mM citrate, Mallinckrodt, Netherlands) was converted to ^67^Ga-chloride (^67^GaCl_3_) [29]. Briefly, ^67^Ga-citrate (82–160 MBq in 2.2 mL) was diluted to 5 mL with dH_2_O and passed through a Silica Light SEP-PAK column (Waters, US) at 1 mL/min. After washing with 5 mL dH_2_O, trapped ^67^Ga was eluted with 0.1 M HCl (Sigma, UK) and collected in 0.5 mL fractions. Fractions with the highest activity concentration ^67^Ga-chloride (200–800 MBq/mL, fractions 6 or 7) were used.

### 2.2 Cell-free DNA damage by ^111^In and ^67^Ga

125 ng pBR322 (10 μL supplied in 10 mM Tris–HCl (pH 8.0) with 1 mM ethylenediaminetetraacetic acid (EDTA), Sigma) was mixed with 1 MBq ^111^In-chloride, ^67^Ga-chloride or ^67^Ga-citrate and incubated up to 72 h at 4 °C (final EDTA concentration 0.1 mM). The final volume was 100 μL in Dulbecco's phosphate-buffered saline pH 7.4 (PBS; Thermo Fisher, UK).

Plasmids were co-incubated with 14 mM dimethylsulfoxide (DMSO), excess EDTA (5 mM) or diethylenetriamine pentaacetic acid (DTPA; 5 mM). Controls included untreated plasmid (no radionuclide), external irradiation (where radionuclides in a 50 mL tube were physically separated from plasmid in 1.5 mL microcentrifuge tube inside the 50 mL tube), and equivalent amounts of non-radioactive gallium- (0.69 pmol) and indium-chloride (0.58 pmol) at molar concentrations equivalent to 1 MBq radionuclide.

After treatment, 12.5 ng plasmid on a 0.8% agarose gel, run at 100 V for 25 min, was visualized with Gel Red™ (Biotium, USA) by UV transilluminator (Gel Doc-it, BioRad, UK).

### 2.3 Analysis of gel electrophoresis images

**Fig. 1:**
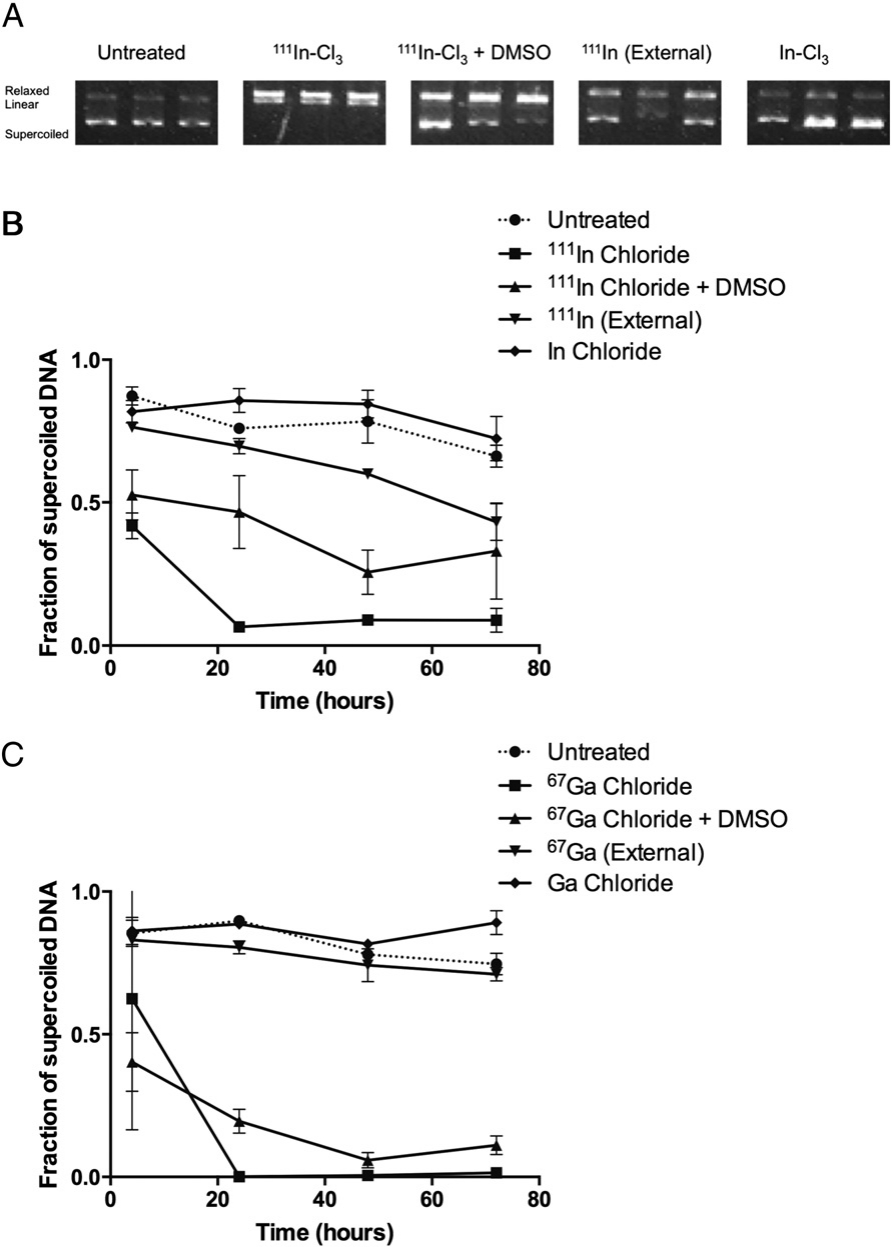
A: Representative image of pBR322 on an agarose gel following treatment with a radionuclide. Here, pBR322 was incubated with 1 MBq ^111^In-chloride (^111^In-Cl_3_ or as external radiation (^111^In (external)) for 72 h in the presence or absence of DMSO or cold indium chloride (InCl_3_). B and C: Fraction of supercoiled (undamaged) plasmid, as measured from gels such as A. Plasmids were incubated with either ^111^InCl_3_ (B) or ^67^GaCl_3_ (C). Data points are average ± standard deviation (SD; n = 2–3). Relaxed bands represent single strand breaks; linear bands are double strand breaks.

**Fig. 2:**
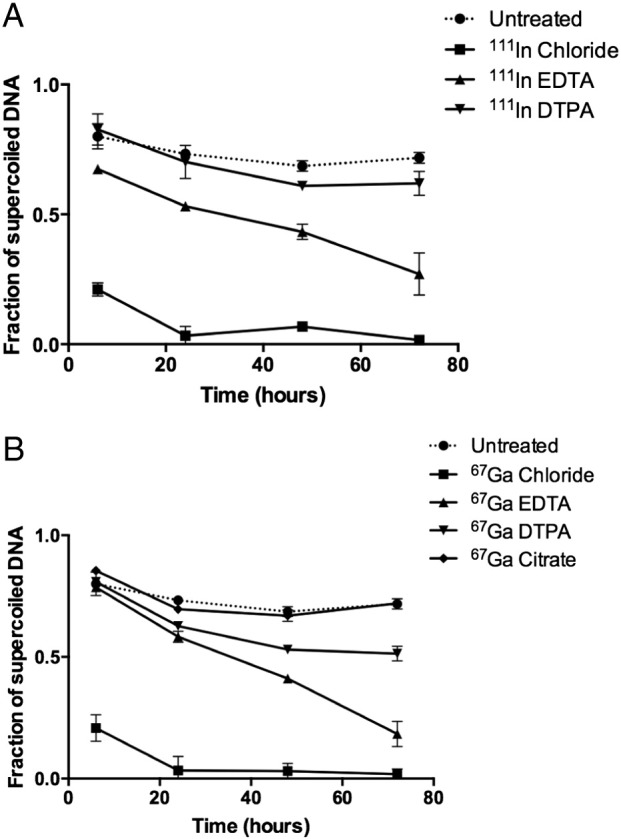
Effect of chelators on plasmid damage treated with 1 MBq ^111^In-chloride (A) or ^67^Ga-chloride (B). The amount of supercoiled DNA (undamaged) was measured from gels where plasmid was incubated with the radionuclide in the presence or absence of chelators EDTA, DTPA and citrate for up to 72 h. Data points are average ± SD (n = 2–3).

Images were analyzed by densitometry of each plasmid band (Fig. 1, Fig. 2, S1–3; supercoiled, circular and linear; Image J 1.48, NIH, USA). Background was measured and subtracted from band intensity. The fraction of supercoiled plasmid (undamaged) of total plasmid represents undamaged plasmid.

### 2.4 ^111^In- and ^67^Ga-oxinate complex synthesis

Lipophilic complexes were prepared from ^67^Ga citrate as described previously [30]. ^67^Ga citrate (Mallinckrodt Medical Inc., Netherlands; 20–50 MBq, 0.1–1 mL) was added to 50 μg oxine (8-hydroxyquinoline; dissolved in ethanol at 1 mg/mL; Sigma). The solution was extracted into dichloromethane (Sigma, USA) and this fraction separated and evaporated to dryness and reconstituted in 2% ethanol in saline.

Tropolone (2-hydroxy-2,4,6-cycloheptatriene-1-one) (Sigma) was dissolved in ethanol (1 mg/mL). MPO (2-mercaptopyridine-N-oxide) (Sigma) was dissolved in distilled water at 1 mg/mL. ^67^Ga citrate (Mallinckrodt Medical Inc., Netherlands) was added at 20–50 MBq (0.1–1 mL) to the ligand solution (50 μg tropolone and 500 μg MPO). The resulting solutions were extracted into dichloromethane (Sigma, USA) and measured for labeling yield following drying.

Up to 75 MBq ^111^In-chloride (0.1–0.2 mL), adjusted to pH 6 with acetate buffer, was added to oxine (1 mg/mL in 2% ethanol) and vortexed for 5 min. ^111^In-oxine complex was extracted into chloroform, evaporated and reconstituted in 2% ethanol in saline [31].

Radiochemical yield of radiometal complexes was measured in a dose calibrator.

### 2.5 Cell culture

Human prostate cancer cells DU145, courtesy of Dr. Florian Kampmeier [32], and breast cancer cells HCC1954, were grown in RPMI-1640 at 37 °C in a humidified atmosphere with 5% CO_2_. Human breast cancer cells MDA-MB-231 were grown in Dulbecco's Modified Eagle Medium (DMEM; with high glucose 4.5 g/L; PAA, Austria). Media were supplemented with L-glutamine (1.5 mM; PAA Laboratories, Austria), 10% fetal bovine serum (Invitrogen) and penicillin (50 I.U./mL)/streptomycin (50 μg/mL) (Invitrogen). Prior to use, cells were trypsinised and washed twice with PBS.

### 2.6 Cellular uptake and retention of radionuclide oxine complexes

Cells (10^6^) in suspension were incubated with 0.1 MBq ^67^Ga- or ^111^In-oxine in 1 mL PBS for 1 h at 37 °C and 5% CO_2,_ then pelleted and washed twice with PBS. Cell-bound (pellet) and free (supernatant) activity was measured by gamma counter.

For cellular retention studies, cells were treated and washed as above and plated in a 6-well plate for three days. At different times, medium was collected, cells washed, and the amount of cell-bound versus free activity measured. The percentage of cell-bound activity retained within the cell at time points after 1 h (set at 100%) was measured.

### 2.7 Viability assay

Cells (2.5 × 10^5^) were incubated with ^67^Ga- (2–25 MBq/mL) or ^111^In-oxine (0.5–30 MBq/mL) in medium (250 μL total) at 37 °C for 1 h. Oxine and ethanol concentrations were 7 μM and 1%, respectively. Controls included ^67^Ga-citrate and ^111^In-chloride (no significant uptake in cells) and decayed oxine complexes at levels equivalent to complete decay of 20 MBq/mL samples.

Following incubation, cells were centrifuged, washed and seeded in medium in 6-well plates for 3 days at 37 °C in 5% CO_2_. Cells were then washed, trypsinised and counted for viability by trypan blue exclusion.

### 2.8 Clonogenic survival

Cells were treated as in the viability assay, but after treatment and washing, 800–2500 cells were seeded in 6-well plates at 37 °C in 5% CO_2_ for 10–14 days. Medium was replaced every 3 days. Colonies (>50 cells) were fixed, stained with methanol/1% crystal violet (Sigma, 1:1) and counted. The results were plotted as the surviving percentage relative to untreated values, with the latter set at 100%.

### 2.9 Statistical analysis

For plasmid studies, data were analyzed by 2-way ANOVA. Student and paired t-tests were used to compare preparations at any one particular time point or the results from the oxine studies, respectively. Statistical analyses were carried out with GraphPad Prism 5 (version 5.04, GraphPad Software Inc., USA).

## 3 Results

### 3.1 Cell free plasmid DNA damage by ^111^In and ^67^Ga

Incubation of pBR322 supercoiled DNA with ^111^In and ^67^Ga (0.1-1 MBq) led to single and double DNA strand breaks, i.e. conversion of supercoiled plasmid to either relaxed or linear plasmid, respectively. Plasmid integrity (i.e. the fraction of plasmid remaining in the supercoiled form) decreased as radioactivity increased; Fig. 1A; Fig. S1). As this activity produced significant damage without assay saturation, it was deemed suitable for comparative studies. DNA damage was detected as early as 4 h post incubation (Fig. 1B–C) and after 24 h of incubation, the supercoiled DNA fractions were reduced to 0.001 ± 0.002 and 0.06 ± 0.01 for ^67^Ga and ^111^In respectively (p = 0.002 compared to untreated). In contrast, untreated controls (0.76 to 0.90) and non-radioactive In-chloride (5.8 nM; 0.89 ± 0.002) or Ga-chloride (6.9 nM; 0.86 ± 0.04) controls produced little DNA damage.

Plasmid DNA was partially protected against ^111^In-induced damage, and less so from ^67^Ga, by co-incubation with DMSO (Figs. 1 and S2). At 24 h, DMSO co-incubation led to supercoiled fractions of 0.47 ± 0.13 and 0.20 ± 0.04, for 1 MBq ^111^In and ^67^Ga, respectively.

External irradiation (i.e. radionuclide separated from the plasmid by the walls of a plastic tube; so that only gamma photons were incident upon the plasmid-containing solution) produced relatively little DNA damage for ^67^Ga (p > 0.05 at 48 h compared to untreated controls). External ^111^In produced significantly more DNA damage than untreated controls (p < 0.05 at 48 h), but significantly less than internal ^111^In-chloride with and without DMSO (p < 0.001 and p < 0.001 at 48 h, respectively).

The addition of chelating agents EDTA, DTPA and citrate provided partial protection of DNA against damage by both radionuclides (Figs. 2, S3). At 72 h, ^67^Ga-chloride plus EDTA or DTPA (5 mM) gave a supercoiled fraction of 0.18 ± 0.05 or 0.51 ± 0.03, respectively, compared to 0.02 ± 0.02 for ^67^Ga-chloride only (Fig. 2B). Similarly, ^67^Ga-citrate produced less DNA damage (supercoiled fraction: 0.72 ± 0.02) than ^67^Ga-chloride. Incubation with ^111^In-chloride plus additional EDTA or DTPA led to supercoiled fractions of 0.27 ± 0.08 or 0.62 ± 0.05, respectively, compared to 0.02 ± 0.02 for ^111^In-chloride alone (Fig. 2A).

### 3.2 Radionuclide oxine synthesis

Radiolabelling yields for ^67^Ga-oxine, -tropolone, and -MPO were 92%, 80%, and 25%, respectively, and 98% for ^111^In-oxine.

### 3.3 Binding and retention of radionuclide oxine complexes

**Fig. 3:**
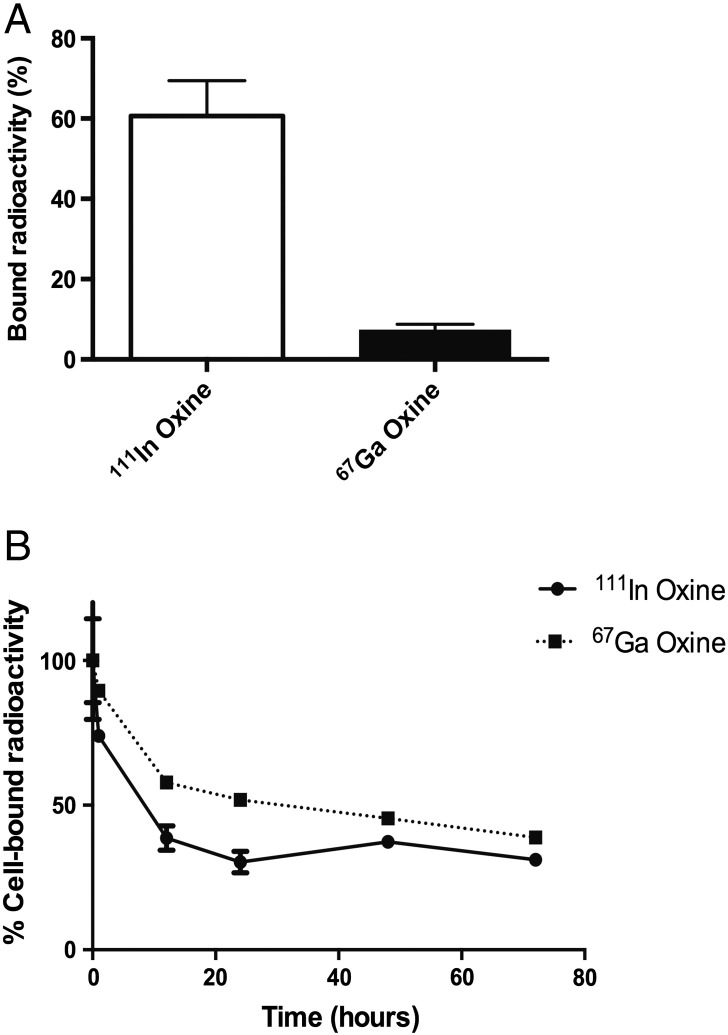
Cell binding (A) and retention (B) of 0.1 MBq ^111^In-oxine and ^67^Ga-oxine in 10^6^ DU145 cells following an initial one-hour incubation. 100% in panel B refers to the maximum bound radioactivity at one hour (A). Data are average ± SD (n = 3/group).

^67^Ga-oxine gave the highest cell binding (Fig. S4); all subsequent studies were carried out with the oxine complex. In DU145 cells, a one-hour incubation with ^111^In-oxine or ^67^Ga-oxine allowed radionuclide binding at 60.6 ± 8.8% or 7.5 ± 1.3%, respectively (Fig. 3A). This decreased with time with only 31.2 ± 1.4% and 38.8% ± 0.7% of the initially-bound ^111^In and ^67^Ga, respectively, retained 72 h after a one-hour incubation period (Fig. 3B). Similar results were found in cell lines MDA-MB-231 and HCC1954 (Figs. S5, S6). The different cell labeling efficiencies of ^111^In-oxine and ^67^Ga-oxine raise the issue of whether to discuss cellular toxicity in relation to the radioactivity to which the cells are exposed in total (i.e. the radioactivity added to the culture) or the radioactivity that is accumulated in the cells (referred to as “cell-bound” activity henceforth). Both are discussed together in the following paragraphs.

### 3.4 Trypan blue viability assay

**Fig. 4:**
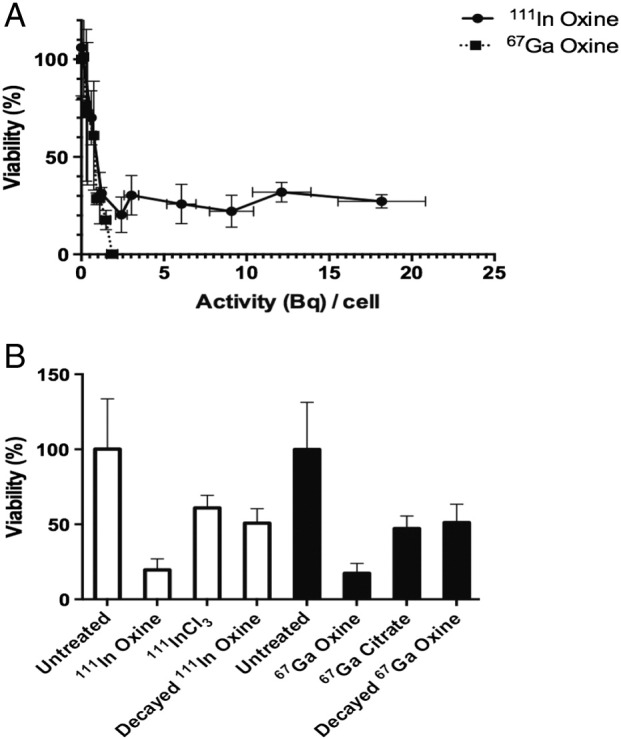
Viability (trypan blue) of DU145 cells treated with ^111^In-oxine or ^67^Ga-oxine at 72 h. A: Viability with increasing cell-bound activities (Bq) per cell. B: Controls for radionuclide oxine treatment: untreated cells, non-cell-bound radioactivity and decayed oxine complexes, standardized at 15 MBq/mL, inducing cellular uptake of 9.09 ± 1.33 Bq/cell for ^111^In-oxine and 1.12 ± 0.20 Bq/cell for ^67^Ga-oxine groups. Data are average ± SD (n = 3/group).

A_50_ is defined as the cell-bound activity causing 50% reduction in viability relative to untreated cells (100%). Three days after an initial one-hour incubation period with ^67^Ga-oxine, the A_50_ was approximately 1 Bq/cell (Table 1, Fig. 4A). Cell-bound ^67^Ga activity required to reduce viability to 17.4 ± 6.6% was approximately 1.5 Bq/cell; this required incubation with 15 MBq/mL ^67^Ga-oxine (Fig. 4B). At this concentration, ^67^Ga-citrate, which was not taken up significantly in cells, caused only 53% loss in viability (Fig. 4B). A similar loss in viability occurred in the control sample incubated with decayed ^67^Ga-oxine.

Qualitatively similar results were obtained with ^111^In; the A_50_ was approximately 1 Bq/cell. However, even at cell-bound activities up to 19 Bq/cell, viability did not drop below 20%. As for ^67^Ga, the controls showed a significant level of toxicity caused by decayed ^111^In-oxine similar to that caused by ^111^In-chloride, which did not bind significantly to cells. Interestingly, ^67^Ga-oxine-induced toxicity at 15 MBq/mL was the same as that caused by the same concentration of ^111^In-oxine, despite this concentration of ^111^In-oxine yielding almost 10-fold higher activity per cell. Non-cell bound ^111^In-chloride caused toxicity (viability around 50%). A similar level of toxicity resulted from the purely chemical effect of decayed ^111^In-oxine.

Qualitatively similar results for both ^67^Ga and ^111^In were found in cell lines MDA-MB-231 and HCC1954 (Figs. S7 and S8 and Table S1).

### 3.5 Clonogenic survival assay

**Fig. 5:**
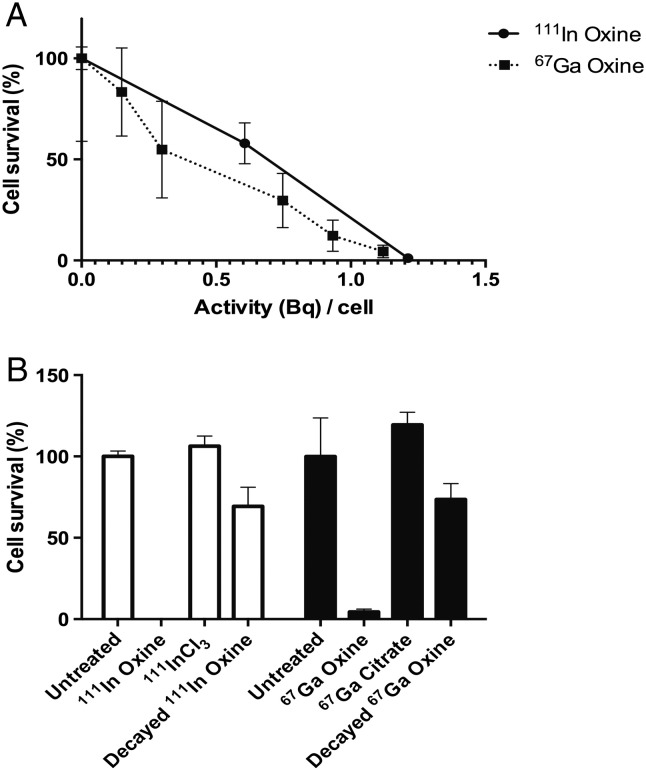
Clonogenic assay of DU145 cells treated with ^111^In-oxine or ^67^Ga-oxine. A: Clonogenicity with increasing cell-bound activities (Bq) per cell. B: Controls for radionuclide oxine treatment: untreated cells, non-cell-bound radioactivity and decayed oxine complexes, standardized to treatment at 15 MBq/mL, achieving uptake of 9.09 ± 1.33 Bq/cell for ^111^In-oxine and 1.12 ± 0.20 Bq/cell for ^67^Ga-oxine groups. Data are average ± SD (n = 3/group). Clonogenicity for ^111^In-oxine at 0.28 ± 0.48% is not visible on the graph.

A one-hour incubation period with ^67^Ga-oxine (15 MBq/mL) with cellular uptake as little as 1.1 Bq/cell was enough to diminish clonogenic survival to 4.4% ± 3.1% compared to untreated controls (Fig. 5A). Replacing ^67^Ga-oxine with ^67^Ga-citrate at this same concentration, with minimal cellular uptake, caused no significant loss in clonogenicity compared to untreated controls (Fig. 5B). Qualitatively similar results were obtained for ^111^In demonstrating that neither radionuclide affected clonogenicity significantly unless bound to the cell (Fig. 5B). Fully decayed radioactive ^67^Ga-oxine and ^111^In-oxine added to the incubation medium led to a significant decrease in relative clonogenic survival (to 74 ± 17% and 69 ± 20% for decayed ^67^Ga-oxine and ^111^In-oxine, respectively, see Fig. 5B) compared to untreated controls. However this chemical toxicity was much less than the toxicity of their non-decayed counterparts, indicating that the radioactivity was by far the major contributor to the observed toxic effect. Qualitatively similar results were found in cell lines MDA-MB-231 and HCC1954 (Figs. S9 and S10 and Table S2).

## 4 Discussion

The plasmid data presented here suggest the involvement of different mechanisms of DNA damage. These include ionization and formation of free radicals along the tracks of Auger electrons, local ionization events caused directly by the residual ion after Auger electron emission (short range effects), free radicals diffusing significant distances from the Auger electron track and residual ion, and minor ionization and free radicals caused by gamma photons (long range effects).

DNA damage produced by ^67^Ga was significantly reduced by chelation with EDTA, DTPA or citrate and incubation with hydroxyl radical scavenger DMSO. The protective effect of chelating the radionuclides with EDTA, DTPA or citrate, on DNA may be a distance effect; assuming unchelated positively-charged In^3+^ and Ga^3+^ bind directly to negatively charged DNA, as has previously been shown for ^111^In [33]. Complexing ^67^Ga with EDTA, forming a negatively charged 1:1 6-coordinate complex, would completely envelope the ^67^Ga atom, preventing metal coordination by plasmid DNA [34]. However, ^111^In forming a 1:1 complex with EDTA would leave some possibility for DNA to coordinate directly to the radiometal which would remain coordinatively unsaturated because of its larger ionic radius [35]. DTPA offers more protection than EDTA against DNA damage by ^111^In (Fig. 2); this may be because being octadentate it more completely fills the coordination sphere of indium than EDTA does [36]. The degree of protection (EDTA < DTPA < citrate_2_) is also in line with the negative charge of the resulting complex: (1-, 2-, 5-).

Free radical scavengers such as DMSO are unlikely to protect against the short-range effects. Previous studies with free radical scavengers have focused on ^125^I, where DMSO reduced strand breakage by 40% if ^125^I was not bound to DNA. When bound to DNA, damage induced by ^125^I was not diminished by DMSO [37]. Also, non-plasmid-bound ^99m^TcO_4_^−^ caused several fold lower induction of single strand breakage in the presence of DMSO [38], [39].

Overall, direct incubation of radionuclides with plasmid DNA in a cell-free system is useful to understand DNA damage by radionuclide emissions and decay only. However, in these experiments radionuclides can directly bind DNA, thus overestimating the potential damage compared to the cellular environment, where direct binding to DNA is less likely.

Results obtained in cell studies showed that ^111^In and ^67^Ga induced high clonogenic toxicity only if incorporated into the cell; external radionuclides and other variables had little effect. Nonetheless decayed radionuclides, producing amongst other compounds zinc and cadmium, did influence both viability and clonogenicity. External irradiation via gamma emissions produced more DNA damage for ^111^In-chloride than ^67^Ga-chloride due perhaps to higher gamma ray exposure rate constants. Surprisingly, cell viability was decreased for non-internalized ^111^In (60.9 ± 8.4%) and ^67^Ga (47.2 ± 8.4%) compared to untreated cells, while clonogenic toxicity was not. This highlights that they measure different aspects of cytotoxicity and are complementary rather than alternative methods.

The clonogenic toxicity of incorporated radioactivity is similar for the two radionuclides and for all three cell lines, with an A_10_ of approximately 1 Bq/cell. This similarity should be interpreted cautiously, since the cellular toxicity of Auger electron emitters is likely to be highly dependent on the intracellular distribution of the radionuclides, which we have not determined and which may not be the same for the two radionuclides. Nevertheless this figure may be a useful guide to how much radioactivity must be incorporated into cancer cells in vivo for effective targeted radionuclide therapy (tRT) and could be used to assess feasibility of clinical tRT.

It should be noted that ^67^Ga-oxine is not a very effective method of incorporating ^67^Ga into cells. Results were, however, consistent with previous trends, including radiolabeling yields for oxine with ^67^Ga [30] and cell labeling numbers of ^111^In-oxine [31] and ^67^Ga-oxine [40]. Efficient cell labeling with ^111^In is probably due to ^111^In-oxine diffusing into the cell cytoplasm and dissociating whereupon ^111^In binds intracellular macromolecules and is trapped within the cell [31]. In leukocytes, ^111^In-oxine also partly localizes to the nucleus [41]. If ^67^Ga-oxine forms a more stable complex [40], the radionuclide may diffuse in but become trapped less readily due to slower dissociation. In order to achieve comparable cellular uptake (Bq/cell), the radioactive concentration of ^67^Ga-oxine was increased compared to ^111^In-oxine.

Future studies should focus on targeted approaches as well as in vivo therapeutic studies comparing ^67^Ga with ^111^In as well as beta emitters such as ^177^Lu. Interestingly, the higher energy Auger electrons emitted by ^67^Ga compared to other Auger electron-emitting radionuclides may provide an advantage by relaxing the requirement for ^67^Ga to be localized in specific sub-cellular compartments (in particular the nucleus) in order to be effective as a therapeutic. The critical observation that ^67^Ga (and similarly ^111^In) has to be cell bound to be effective suggests that future targeting studies can focus on the feasibility in vivo of achieving target uptake of around 1 Bq per cancer cell required for effective cell killing.

## 5 Conclusion

^67^Ga damages plasmid DNA in a manner that may be dependent on distance to the DNA, which in turn may be affected by the chemical form of the radionuclide. Neither ^67^Ga nor ^111^In showed substantial toxicity unless incorporated into the cells. The threshold cellular uptake of ^67^Ga to achieve substantial cell kill is of the order of 1 Bq per cell. ^67^Ga deserves further evaluation for radionuclide therapy, especially in the context of a theranostic pairing with ^68^Ga.

## Figures and Tables

**Table 1:** Cell-bound activity per cell (Bq/cell) required for a 50% (A_50_) and 90% (A_10_) reduction in viability and clonogenicity in DU145 cells compared to untreated cells (determined by interpolation of data in Figs. 4A and 5A).

	A_50_ (Bq/cell)		A_10_ (Bq/cell)	
	^67^Ga-oxine	^111^In-oxine	^67^Ga-oxine	^111^In-oxine
Viability (Trypan blue)	1.0	1.0	1.5	N/A
Clonogenic	0.3	0.7	1.0	0.9

No A_10_ value exists for ^111^In-oxine (viability assay), as loss of membrane integrity was not achieved in more than 75% of cells.

## Appendix A Supplementary data

Supplementary material Image 1
