# Supplementary material for: Re-assessing gallium-67 as a therapeutic radionuclide
Source: Nucl Med Biol. 2017 Mar;46:12–8. doi: 10.1016/j.nucmedbio.2016.10.008 (PMC5303015; doi:10.1016/j.nucmedbio.2016.10.008)
Supplement: Supplementary file 1 — Supplementary material [file mmc1.docx]

**Appendix**

**Figure S1.** Imaging **(A)** and quantification **(B)** of plasmid damage assessment following incubation with 0.1, 0.5 and 1 MBq of ^111^In-chloride incubation at 4 and 24 hours. Supercoiled DNA refers to undamaged plasmid. Data are average ± standard deviation (n=3/group).


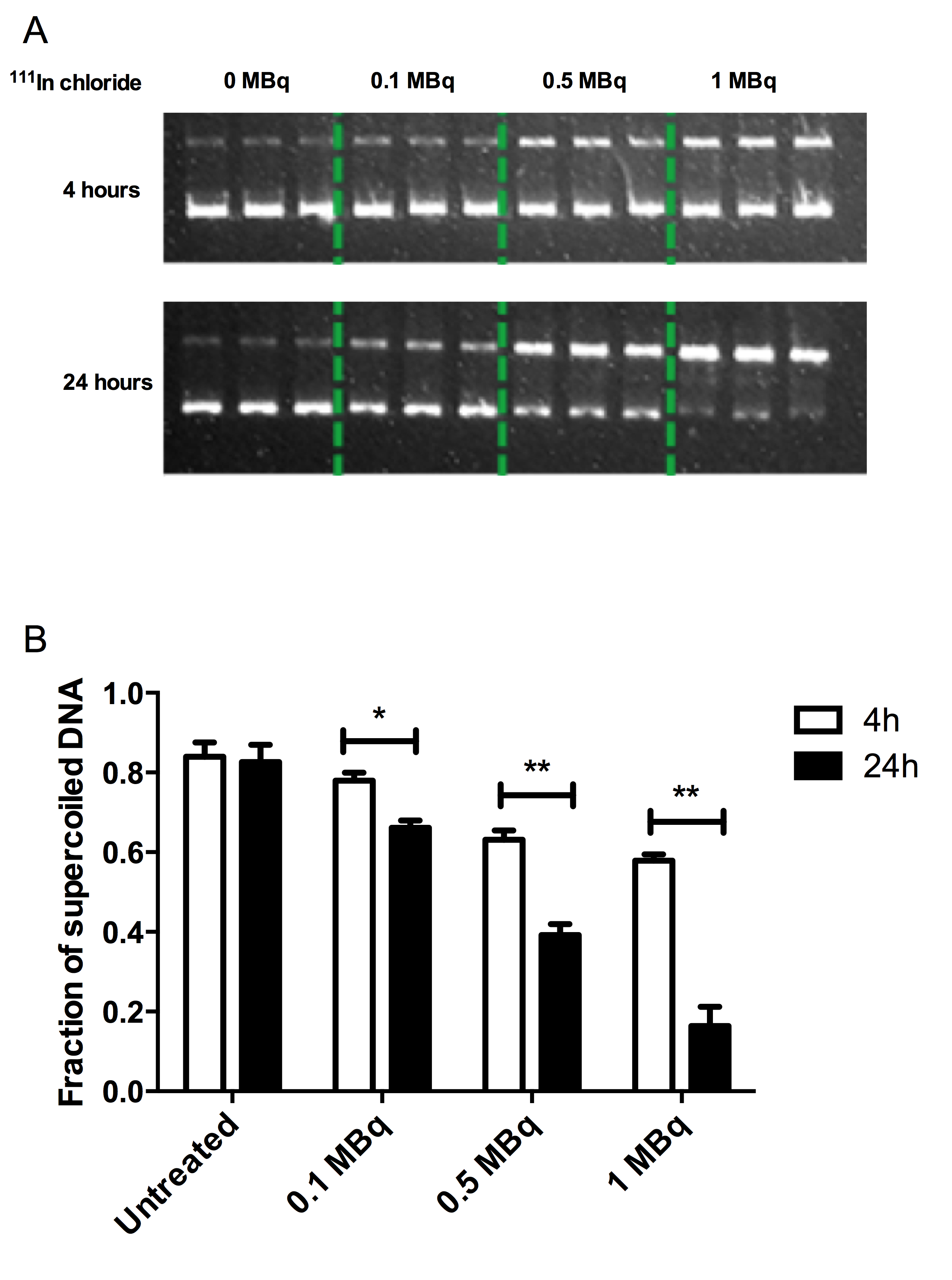


**Figure S2.** Images of plasmid following treatment with 1 MBq ^67^Ga-chloride **(A)** or ^111^In-chloride **(B)** at 4, 24, 48 and 72 hours of incubation in the presence or absence of DMSO. Controls include untreated plasmid, external irradiation with ^67^Ga- or ^111^In-chloride and non-radioactive Ga- or In- chloride and In-Cl.

A

B

**Figure S3.** Images of plasmid following treatment with 1 MBq ^67^Ga-chloride **(A)** or ^111^In-chloride **(B)** at 6, 24, 48 and 72 hours of incubation in the presence or absence of chelators EDTA (5 mM), DTPA (5 mM) or citrate (0.9-1.7 mM).

A

B

**Figure S4.** Cell binding study with ^67^Ga-labeled lipophilic complexes, namely oxine, tropolone and MPO, after a one-hour incubation at 37 ͦC in 5% CO_2_ incubator in MDA-MB-231 cells (1 x 10^6^ cells/mL in 1 mL Dulbecco's PBS). Cells were pelleted and the supernatant (free activity) and pellets (bound) were separately measured for radioactivity using a gamma counter (Wallac, Germany). Data are average ± standard deviation (n=2/group).

**Figure S5.** Cell binding of 0.1 MBq ^111^In-oxine and ^67^Ga-oxine in 1 x 10^6^ MDA-MB-231 or HCC1954 cells in 1 mL following an initial one-hour incubation. Data are average ± standard deviation (n=3/group).

**Figure S6.** Retention of 0.1 MBq ^111^In-oxine and ^67^Ga-oxine in 1 x 10^6^ MDA-MB-231 **(A)** or HCC1954 **(B)** cells in 1 mL following an initial one-hour incubation. Data are average ± standard deviation (n=3/group).

**Figure S7.** Viability trypan blue assay of MDA-MB-231 cells (in 1 mL) following treatment with ^111^In-oxine or ^67^Ga-oxine (20 MBq/mL). Viability was measured at 72 hours after incubation of cells with the radionuclides. **A:** Viability with increasing activities (Bq) per cell. **B:** Controls for radionuclide oxine treatment including untreated cells, non-internalised radioactivity and decayed oxine complexes. Data are average ± standard deviation (n=3/group).

**Figure S8.** Viability trypan blue assay of HCC1954 cells (in 1 mL) following treatment with ^111^In-oxine or ^67^Ga-oxine (20 MBq/mL). Viability was measured at 72 hours after incubation of cells with the radionuclides. **A:** Viability with increasing activities (Bq) per cell. **B:** Controls for radionuclide oxine treatment including untreated cells, non-internalised radioactivity and decayed oxine complexes. Data are average ± standard deviation (n=3/group).

**Figure S9.** Clonogenic assay of MDA-MB-231 cells (in 1 mL) following treatment with ^111^In-oxine or ^67^Ga-oxine (20 MBq/mL). **A:** Clonogenicity i.e. surviving fraction with increasing activities (Bq) per cell. **B:** Controls for radionuclide oxine treatment including untreated cells, non-internalised radioactivity and decayed oxine complexes. Data are average ± standard deviation (n=3/group).

**Figure S10.** Clonogenic assay of HCC1954 cells (in 1 mL) following treatment with ^111^In-oxine or ^67^Ga-oxine (20 MBq/mL). **A:** Clonogenicity i.e. surviving fraction with increasing activities (Bq) per cell. **B:** Controls for radionuclide oxine treatment including untreated cells, non-internalised radioactivity and decayed oxine complexes. Data are average ± standard deviation (n=3/group).

**Table S1.** Activity per cell (Bq/cell) required for a 50% and 90% reduction in viability in MDA-MB-231 and HCC1954 cells.

| Cells | A_50_ (Bq/cell) | | A_10_ (Bq/cell) | |
| --- | --- | --- | --- | --- |
|  | ^67^Ga | ^111^In | ^67^Ga | ^111^In |
| MDA-MB-231 | 1 | 0.5 | 2 | - |
| HCC1954 | 0.5 | 2.5 | 1 | - |

**Table S2.** Activity per cell (Bq/cell) required for a 50% and 90% reduction in clonogenicity in MDA-MB-231 and HCC1954 cells.

| Cells | A_50_ (Bq/cell) | | A_10_ (Bq/cell) | |
| --- | --- | --- | --- | --- |
|  | ^67^Ga | ^111^In | ^67^Ga | ^111^In |
| MDA-MB-231 | 0.25 | 0.15 | 1.55 | 0.875 |
| HCC1954 | 0.1 | 0.6 | 0.5 | 0.8 |
